# Cigarette smoking and cessation indicators by glycemic status among adults in the United States, 2011–2023

**DOI:** 10.1016/j.pmedr.2026.103597

**Published:** 2026-08-19

**Authors:** Fuzhuo Wang, Jiashuang Xu, Yuxuan Ma, Jason Wong, Ligang Liu, Ye Huang

**Affiliations:** a Department of General Surgery, The First Affiliated Hospital of Jinzhou Medical University, Jinzhou, Liaoning Province, China; b Nursing Department, Peking Union Medical College Hospital, Chinese Academy of Medical Science and Peking Union Medical College, Beijing, China; c The Fourth School of Clinical Medicine, China Medical University, Shenyang, Liaoning Province, China; d Department of Pharmacy Practice and Administration, College of Pharmacy, Western University of Health Sciences, Pomona, California, USA

**Keywords:** National Health and nutrition examination survey, Cigarette smoking, Smoking cessation, Quit ratio, Quit attempts, Prediabetes, Glycemic control, Diabetes

## Abstract

**Objective:**

To compare smoking prevalence, smoking intensity, quit attempts, and quit ratio across four glycemic groups among US adults.

**Methods:**

We analyzed National Health and Nutrition Examination Survey data collected in the United States from 2011 through 2023 among adults aged 20 years or older. Glycemic status was classified as no diabetes, prediabetes, controlled diabetes, or uncontrolled diabetes. Survey-weighted analyses estimated smoking outcomes and adjusted associations using models adjusted for demographic, socioeconomic, behavioral, and clinical factors.

**Results:**

Among 29,096 adults, current smoking prevalence was 17.45%, 19.63%, 14.30%, and 19.83% across the four groups, respectively (*P* < 0.01). Smoking intensity differed across groups (P<0.01). Quit attempt prevalence did not differ significantly (*P* = 0.06), whereas quit ratio differed (56.36%, 57.20%, 71.28%, and 59.69%; *P* < 0.01). In fully adjusted models, prediabetes (adjusted odds ratio [aOR]: 1.31; 95% confidence interval [CI]: 1.10, 1.57) and uncontrolled diabetes (aOR: 1.37; 95% CI: 1.02, 1.83) were associated with higher odds of current smoking. Prediabetes was associated with a lower quit ratio among ever smokers (aOR: 0.80; 95% CI: 0.66, 0.98).

**Conclusions:**

Smoking burden remained substantial among adults with prediabetes and uncontrolled diabetes, supporting targeted cessation interventions across the glycemic spectrum and informing future public health planning.

## Highlights

• Smoking remained common across the glycemic spectrum in United States adults.
• Prediabetes was linked to higher odds of current cigarette smoking.
• Smokers with diabetes reported greater smoking intensity and heavy smoking.
• Prediabetes was associated with a lower quit ratio among ever smokers.
• Findings support targeted cessation care in diabetes prevention and management.

## 1 Introduction

Cigarette smoking remains a leading modifiable cause of cardiovascular disease (CVD) and premature mortality (Rahman et al., 2025; Tasdighi et al., 2025). Its effects are especially important for adults with dysglycemia because smoking has been associated with insulin resistance, impaired glucose metabolism, and elevated cardiovascular risks (Hong et al., 2015; Vlassopoulos et al., 2013; Bergman et al., 2012). For people with diabetes, continued smoking further increases cardiovascular risks, whereas cessation reduces morbidity and mortality (Campagna et al., 2019; Pan et al., 2015). Accordingly, Clinical guidance therefore recommends routine tobacco use assessment and evidence-based cessation treatment as part of diabetes prevention and management (Diabetes⁎, A.D.A.P.P.C.f., 2025a; Russo et al., 2025).

National surveillance has shown that cigarette smoking remains common among adults with diabetes and impaired glucose metabolism. In National Health and Nutrition Examination Survey (NHANES) data from 1999 to 2008, the prevalence of current smoking was 25.7% among adults with diabetes, 24.2% among adults with impaired fasting glucose, and 24.1% among adults without diabetes (Clair et al., 2013). However, most population-based analyses have treated diabetes as a binary condition, comparing adults with and without diabetes. This approach may obscure clinically meaningful heterogeneity across the glycemic spectrum. Prediabetes represents an important prevention window, whereas diabetes with hemoglobin A1c (HbA1c) ≥7.0% may identify a subgroup in which continued tobacco exposure compounds an elevated cardiometabolic burden (Almomani and Al-Tawalbeh, 2022; Chen and Lin, 2025). Distinguishing no diabetes, prediabetes, controlled diabetes, and uncontrolled diabetes may therefore improve surveillance and help identify groups requiring more intensive cessation support.

Smoking prevalence alone does not fully characterize tobacco burden. Smoking intensity may reflect nicotine dependence and treatment need, whereas past-year quit attempts reflect recent cessation activity, and the quit ratio reflects cumulative cessation among ever smokers. Examining these outcomes together can clarify whether differences across glycemic groups arise from heavier exposure, fewer quit attempts, or lower cumulative cessation. Trend analyses further show whether outcomes are improving, stable, or worsening over time. Such monitoring can identify groups not benefiting equally from tobacco-control efforts, guide prevention and treatment resources, and support evaluation of future interventions. Updated surveillance is needed because earlier studies predated major changes in tobacco use and healthcare delivery, including disruptions associated with the coronavirus disease 2019 pandemic (Clair et al., 2013; Fan et al., 2013; Koonin et al., 2020) (Bandi et al., 2022).

To address these gaps, we used serial cross-sectional NHANES data from 2011 through 2023 to compare current smoking, smoking intensity, past-year quit attempts, and quit ratio across four glycemic groups and to evaluate trends across survey cycles. Because demographic, socioeconomic, behavioral, and health-related characteristics are associated with both glycemic status and smoking, sequential survey-weighted regression models assessed whether group differences persisted after adjustment for potential confounders. We hypothesized that adults with prediabetes and those with uncontrolled diabetes would have a higher prevalence of current smoking and less favorable cessation indicators than adults without diabetes.

## 2 Methods

### 2.1 Study design and population

We conducted a serial cross-sectional analysis of five NHANES periods: 2011–2012, 2013–2014, 2015–2016, 2017-March 2020, and August 2021–August 2023. NHANES represent the civilian, noninstitutionalized population of the United States. Data are collected through household interviews, standardized examinations, and laboratory testing. This secondary analysis used de-identified, publicly available data and was therefore exempt from additional institutional review board approval. Interview and examination response rates declined from 72.64% and 69.53%, respectively, in 2011–2012 to 34.5% and 25.6% in August 2021–August 2023; cycle-specific response rates and analytic sample sizes are provided in Table S1.

**Fig. 1:**
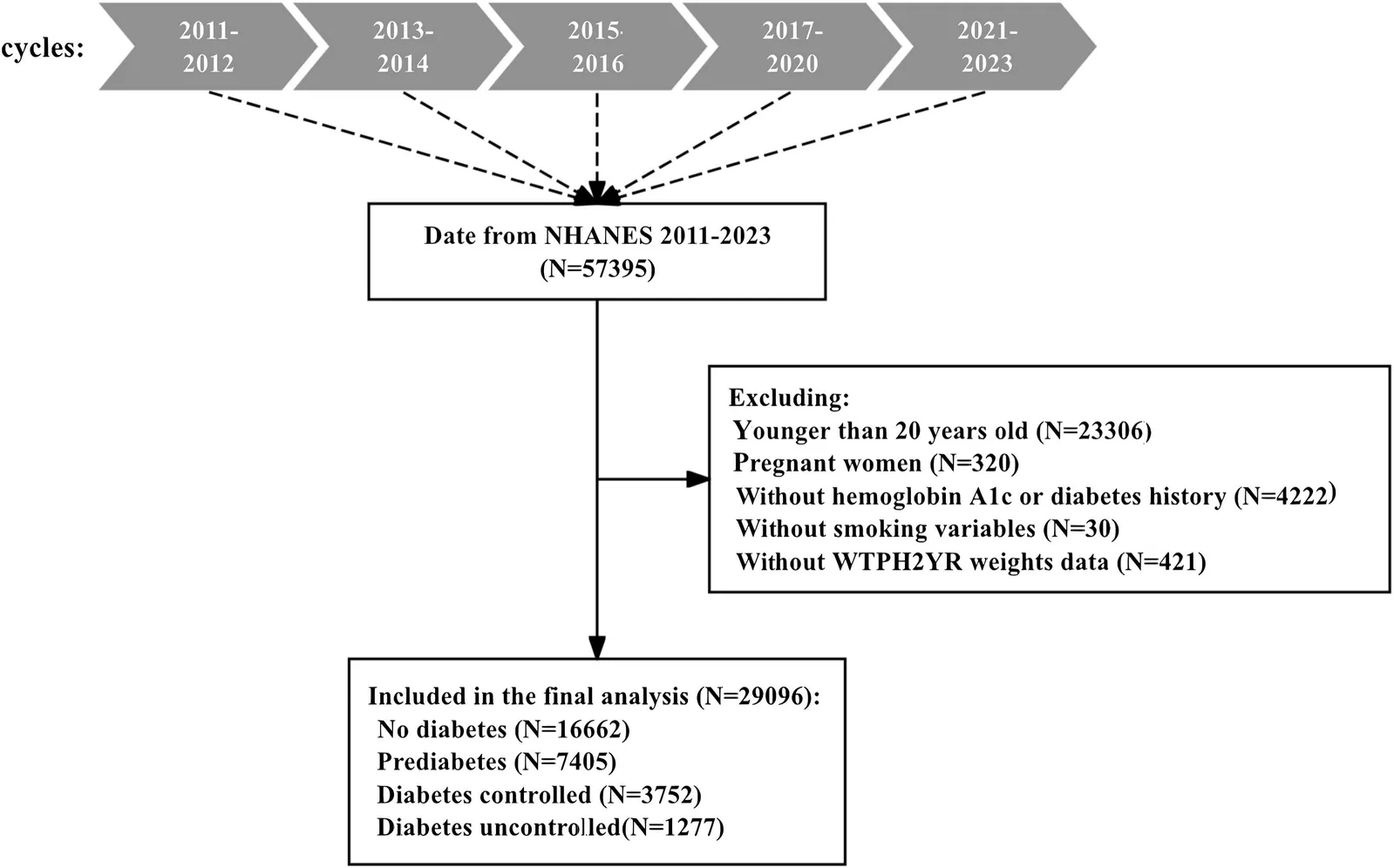
Flow chart of the population included in the study.

Participants were eligible if they were aged 20 years or older and had non-missing information on HbA1c, diabetes history, and cigarette smoking status. Among 57,395 participants, we excluded 23,306 who were younger than 20 years, 320 who were pregnant, 4222 with insufficient glycemic information, 30 with insufficient smoking information, and 421 without the required survey weight. The final analytic sample included 29,096 adults: 16,662 with no diabetes, 7405 with prediabetes, 3752 with diabetes and HbA1c below 7.0%, and 1277 with diabetes and HbA1c of 7.0% or higher. Participant selection is shown in Fig. 1.

### 2.2 Measures

#### 2.2.1 Glycemic status

Glycemic status was determined using self-reported physician-diagnosed diabetes and laboratory-measured HbA1c. Participants were asked whether a doctor or other health professional had ever told them that they had diabetes. Those answering “yes,” or those without self-reported diabetes who had HbA1c of 6.5% or higher, were classified as having diabetes. HbA1c was measured from blood specimens collected during the examination using standardized NHANES laboratory procedures. Among participants without diabetes, no diabetes was defined as HbA1c below 5.7%, and prediabetes as HbA1c from 5.7% to 6.4% (Diabetes⁎, A.D.A.P.P.C.f., 2025b). Among participants with diabetes, glycemic control was defined using HbA1c < 7.0% (controlled) versus ≥7.0% (uncontrolled), consistent with guideline targets for nonpregnant adults (Diabetes⁎, A.D.A.P.P.C.f., 2025c).

#### 2.2.2 Smoking outcomes

Smoking measures were obtained from the NHANES smoking questionnaire and harmonized across survey cycles. Participants reporting fewer than 100 lifetime cigarettes were classified as never smokers. Those reporting at least 100 lifetime cigarettes were classified as ever smokers and were further categorized as current smokers if they smoked every day or some days and former smokers if they smoked not at all.

Among current smokers, smoking intensity was measured using self-reported cigarettes smoked per day, and heavy smoking was defined as ≥20 cigarettes per day (Pierce et al., 2011). A past-year quit attempt was defined as reporting any attempt to stop smoking during the previous 12 months among current smokers. Because NHANES is cross-sectional and does not measure sustained abstinence or relapse over time, we also calculated the quit ratio among ever smokers, defined as the number of former smokers divided by the combined number of former and current smokers (Weinberger et al., 2020).

#### 2.2.3 Covariates

Covariates were selected a priori based on their established relationships with both glycemic status and smoking behavior (Zhang et al., 2026). Demographic covariates included age group (20–39, 40–59, ≥60 years), sex, and race/ethnicity (non-Hispanic White, non-Hispanic Black, Hispanic, Asian, other/multiracial). Socioeconomic covariates included education level (<high school, high school, some college, college graduate or higher), income-to-poverty ratio (categorized as<1.0, 1.0–3.99, ≥4.0), and health insurance (insured vs uninsured).

Body mass index was calculated from measured height and weight and categorized as below 25.0, 25.0–29.9, or 30.0 kg/m^2^ or higher. Depressive symptoms were defined as a Patient Health Questionnaire-9 score of 10 or higher (Kroenke et al., 2001). Alcohol consumption was categorized as never, mild, moderate, or heavy using sex-specific thresholds (Shmulewitz et al., 2021). Cardiometabolic comorbidity was defined by the presence of hypertension, hypercholesterolemia, or self-reported cardiovascular disease. Complete variable definitions and source variables are provided in Table S2.

### 2.3 Statistical analysis

Survey-weighted logistic regression models examined associations between glycemic status and smoking outcomes using sequential adjustment. For current smoking (yes vs no) in the full adult sample, quit attempt (yes vs no) among current smokers, and quit ratio among ever smokers, three models were fitted: Model 1 (unadjusted), Model 2 (adjusted for age, sex, race/ethnicity, educational attainment, income-to-poverty ratio, and insurance status), and Model 3 (Model 2 plus BMI, alcohol use, depressive symptoms, and cardiometabolic comorbidity). Glycemic status (no diabetes as the reference category) was the primary exposure, and results are reported as adjusted odds ratios (aORs) with 95% confidence intervals (CIs).

Smoking prevalence, quit attempts, and quit ratio were age standardized to the 2000 United States standard population. Survey-period midpoint years were entered as continuous variables in survey-weighted models to test linear trends using design-adjusted Wald statistics. Analyses used complete cases for the outcome and covariates included in each model. All statistical tests were two-sided with α = 0.05. All analyses were conducted in R (version 4.2.0).

## 3 Results

### 3.1 Study population and participant characteristics

The analytic sample included 29,096 adults: 16,662 with no diabetes, 7405 with prediabetes, 3752 with controlled diabetes, and 1277 with uncontrolled diabetes. Adults with diabetes were older than those without diabetes (*P* < 0.01). Lower educational attainment, lower income, obesity, cardiometabolic comorbidity, and depressive symptoms were generally more prevalent among adults with diabetes (Table 1).

**Table 1:** Weighted characteristics of adults aged 20 years or older by glycemic status, United States, 2011–2023.

Characteristic	No diabetes	Prediabetes	Controlled diabetes	Uncontrolled diabetes	*P*-value
**Age, years, mean (SE)**	43.40 (0.29)	56.00 (0.32)	61.10 (0.34)	57.00 (0.54)	<0.01
**Age group, n (%)**					<0.01
20–39	7447 (46.65)	1151 (16.86)	163 (6.39)	137 (12.04)	
40–59	5287 (34.49)	2634 (39.06)	1129 (35.77)	482 (43.84)	
≥60	3928 (18.86)	3620 (44.08)	2460 (57.83)	658 (44.12)	
**Female, n (%)**	8815 (52.69)	3852 (52.64)	1783 (45.78)	670 (53.80)	<0.01
**Race/ethnicity, n (%)**					<0.01
Non-Hispanic White	7716 (67.59)	2659 (58.18)	1303 (59.17)	402 (51.81)	
Non-Hispanic Black	2775 (8.42)	2036 (15.92)	979 (14.68)	330 (16.05)	
Hispanic	3588 (15.07)	1626 (15.62)	926 (15.41)	370 (20.94)	
Asian	1848 (5.32)	815 (6.32)	379 (5.92)	123 (6.03)	
Other / Multiracial	735 (3.61)	269 (3.96)	165 (4.82)	52 (5.17)	
**Education, n (%)**					<0.01
<High school	2570 (10.36)	1690 (16.64)	1081 (19.39)	377 (22.11)	
High school/GED	3503 (22.13)	1780 (26.51)	888 (27.78)	299 (26.50)	
Some college	5365 (31.92)	2168 (29.55)	1090 (31.41)	378 (31.93)	
College graduate+	5217 (35.60)	1759 (27.30)	690 (21.42)	219 (19.47)	
**Income-to-poverty ratio, n (%)**					<0.01
Low	2899 (13.45)	1321 (14.67)	790 (16.76)	294 (20.06)	
Moderate	7486 (46.56)	3535 (51.66)	1827 (53.38)	633 (51.43)	
High	4589 (40.98)	1664 (33.67)	688 (29.66)	215 (28.51)	
**Health insurance, n (%)**	13,456 (84.14)	6227 (86.82)	3426 (93.35)	1025 (83.20)	<0.01
**BMI (kg/m**^**2**^**), mean (SE)**	28.16(0.10)	30.89(0.15)	33.15(0.19)	33.46(0.30)	<0.01
**BMI category, n (%)**					<0.01
<25 (normal/underweight)	5842 (34.29)	1497 (19.23)	476 (10.81)	168 (11.79)	
25–29.9 (overweight)	5436 (33.82)	2446 (33.19)	1044 (26.45)	330 (23.00)	
≥30 (obese)	5181 (31.90)	3385 (47.58)	2128 (62.74)	757 (65.21)	
**Cardiometabolic comorbidity, n (%)**	6799 (39.24)	4761 (64.31)	3331 (88.12)	959 (76.59)	<0.01
**Depression, n (%)**	1345 (8.18)	585 (8.71)	493 (13.30)	155 (12.56)	<0.01
**Alcohol use, n (%)**					<0.01
Never	2490 (14.03)	1497 (21.81)	848 (27.23)	294 (25.07)	
Mild	4884 (37.66)	2231 (42.96)	1017 (45.47)	336 (39.11)	
Moderate	4120 (33.13)	1287 (24.21)	463 (18.26)	198 (23.62)	
Heavy	2026 (15.19)	579 (11.02)	229 (9.04)	96 (12.20)	

Note. Values are unweighted counts with survey-weighted percentages unless otherwise indicated; age and body mass index are presented as survey-weighted means with standard errors. Counts may not amount to group totals because of missing data, and percentages may not amount to 100 because of rounding. Controlled diabetes was defined as diabetes with hemoglobin A1c below 7.0%, and uncontrolled diabetes as diabetes with hemoglobin A1c of 7.0% or higher. Income-to-poverty ratio categories were low (<1.0), moderate (1.0–3.99), and high (≥4.0).

Cardiometabolic comorbidity was defined as hypertension, hypercholesterolemia, or a history of cardiovascular disease. Depressive symptoms were defined as a Patient Health Questionnaire-9 score of 10 or higher. Alcohol-use categories were based on sex-specific thresholds described in the Methods. *P* values were obtained from design-adjusted Wald tests accounting for survey weights, strata, and primary sampling units.

### 3.2 Smoking and cessation intensity

Current smoking prevalence differed across glycemic groups (all *P* < 0.01; Table 2): 17.45% among adults without diabetes, 19.63% among those with prediabetes, 14.30% among those with controlled diabetes, and 19.83% among those with uncontrolled diabetes. In Bonferroni-adjusted pairwise comparisons, current smoking prevalence was lower in controlled diabetes than in no diabetes (difference, −3.16%; P<0.01), prediabetes (difference, −5.33%; *P* < 0.01), and uncontrolled diabetes (difference, −5.53%; P<0.01). No other pairwise differences were statistically significant (Table S3).

**Table 2:** Weighted cigarette smoking prevalence, intensity, quit attempts, and quit ratio by glycemic status among adults aged 20 years or older, United States, 2011–2023.

Smoking outcomes	No diabetes	Prediabetes	Controlled diabetes	Uncontrolled diabetes	*P*-value
Current smoker, % (SE)	17.45 (0.63)	19.63 (0.78)	14.30 (0.78)	19.83 (1.30)	<0.01
Cigarettes/day, mean (SE)	11.41 (0.33)	12.08 (0.32)	13.95 (0.79)	13.62 (0.84)	<0.01
Heavy smoking, % (SE)	25.79 (1.53)	28.83 (1.54)	38.13 (3.29)	36.64 (4.76)	<0.01
Quit attempt, % (SE)	49.87 (1.60)	54.57 (2.72)	47.14 (3.81)	41.76 (3.87)	0.06
Quit ratio, % (SE)	56.36 (1.17)	57.20 (1.31)	71.28 (1.39)	59.69 (2.53)	<0.01

Values are survey-weighted to represent adults in the United States and are presented as % (SE) unless otherwise indicated. Current smoking was assessed among all adults. Cigarettes smoked per day, heavy smoking, and past-year quit attempts were assessed among current smokers. Quit ratio was assessed among ever smokers and was calculated as former smokers divided by the sum of former and current smokers.

Among current smokers, cigarette consumption per day was 11.41, 12.08, 13.95, and 13.62 cigarettes per day across the four groups, respectively (P<0.01). The prevalence of heavy smoking among current smokers was 25.79%, 28.83%, 38.13%, and 36.64%, respectively (*P* < 0.01). Heavy smoking was more prevalent in controlled diabetes than in no diabetes (difference, 12.34%; P<0.01) and prediabetes (difference, 9.30%; *P* = 0.04). Other Bonferroni-adjusted comparisons were not statistically significant (Table S3).

Past-year quit-attempt prevalence among current smokers was 49.87% among adults without diabetes, 54.57% in prediabetes, 47.14% in controlled diabetes, and 41.76% in uncontrolled diabetes. The overall difference was not statistically significant (*P* = 0.06). Bonferroni-adjusted comparison indicated that quit attempt prevalence was lower in uncontrolled diabetes than in prediabetes (difference, −12.81%; *P* = 0.04); the remaining pairwise comparisons were not statistically significant (Table S3).

The quit ratio differed significantly across groups (*P* < 0.01): 56.36% in no diabetes, 57.20% in prediabetes, 71.28% in controlled diabetes, and 59.69% in uncontrolled diabetes. Bonferroni-adjusted comparisons showed that the quit ratio was higher in controlled diabetes than in no diabetes (difference, 14.92%; *P* < 0.01) and prediabetes (difference, 14.08%; *P* < 0.01). The quit ratio was lower in uncontrolled diabetes than in controlled diabetes (difference, −11.59%; *P* < 0.01). No other pairwise differences were statistically significant (Table S3).

### 3.3 Temporal trends

Age-standardized smoking prevalence was 17.04%, 21.67%, 19.54%, and 24.62% across the four glycemic groups, respectively (*P* < 0.01). The age-standardized estimates were 48.43%, 56.60%, 47.50%, and 43.97% for past-year quit attempts (*P* = 0.03) and 56.67%, 49.22%, 59.13%, and 48.65% for quit ratio (*P* < 0.01) (Table 3). Bonferroni-adjusted pairwise comparisons are presented in Table S4.

**Table 3:** Age-standardized Smoking Prevalence, Quit Attempts, and Quit Ratio by Glycemic Group among adults aged 20 years or older, United States, 2011–2023.

Smoking outcomes	No diabetes	Prediabetes	Controlled diabetes	Uncontrolled diabetes	P-value
Current smoking, % (SE)	17.04 (0.63)	21.67 (1.02)	19.54 (1.92)	24.62 (2.26)	<0.01
Quit attempts, % (SE)	48.43 (1.70)	56.60 (2.96)	47.50 (5.85)	43.97 (4.99)	0.03
Quit ratio, % (SE)	56.67 (1.13)	49.22 (1.66)	59.13 (3.23)	48.65 (3.44)	<0.01

Note. Values are survey-weighted percentages with standard errors and were directly standardized to the 2000 United States standard population using the prespecified age groups. Current smoking was assessed among all adults, past-year quit attempts were assessed among current smokers, and quit ratio was assessed among ever smokers. Quit ratio was calculated as the number of former smokers divided by the combined number of former and current smokers. Controlled diabetes was defined as diabetes with hemoglobin A1c below 7.0%, and uncontrolled diabetes as diabetes with hemoglobin A1c of 7.0% or higher.

Abbreviations: SE, standard error.

**Fig. 2:**
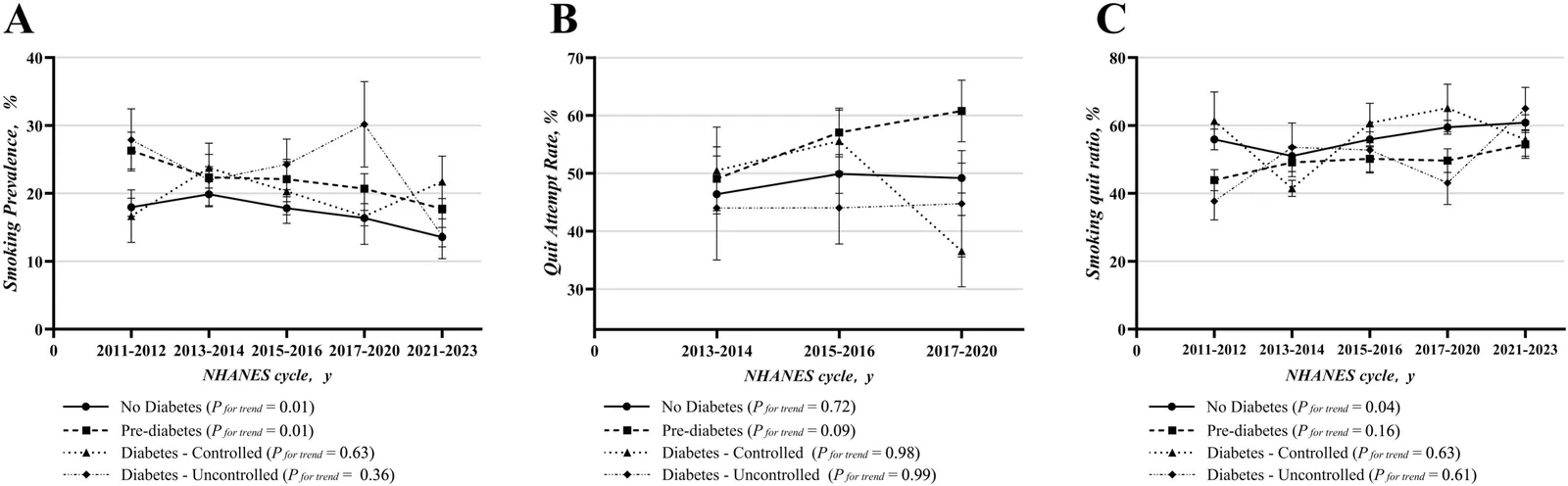
Age-standardized trends in cigarette smoking and cessation indicators across four glycemic status groups among adults aged 20 years or older, United States, 2011–2023. A. Current smoking prevalence among all adults, 2011–2012 through August 2021–August 2023; B. Past-year quit attempts among current smokers, 2013–2014 through 2017-March 2020; C. Quit ratio among ever smokers, 2011–2012 through August 2021–August 2023. Estimates were survey weighted and directly standardized to the 2000 United States standard population. The quit ratio was calculated as the number of former smokers divided by the combined number of former and current smokers. Controlled diabetes was defined as diabetes with hemoglobin A1c below 7.0%, and uncontrolled diabetes as diabetes with hemoglobin A1c of 7.0% or higher.

Age-standardized current smoking prevalence declined from 17.96% in 2011–2012 to 13.56% in August 2021–August 2023 among adults without diabetes (P for trend = 0.01) and from 26.29% to 17.74% among those with prediabetes (P for trend = 0.01). No significant trends were observed among adults with controlled diabetes (*P* = 0.63) or uncontrolled diabetes (*P* = 0.35; Fig. 2 and Table S5). No significant trends in quit attempts were observed within any glycemic group. The quit ratio increased from 55.90% to 60.82% among adults without diabetes (P for trend = 0.04), whereas trends were not significant in the other groups.

### 3.4 Multivariable associations between glycemic status and outcomes

Glycemic status remained associated with current smoking in the fully adjusted model (P<0.01; Table 4). Compared with adults without diabetes, those with prediabetes had higher odds of current smoking (aOR 1.31, 95% CI: 1.10, 1.57) (Table 4). Uncontrolled diabetes was associated with higher odds in the fully adjusted model (aOR 1.37, 95% CI: 1.02–1.83), whereas controlled diabetes was not significant after full adjustment (aOR 0.95, 95% CI: 0.76, 1.19).

**Table 4:** Survey-weighted associations of glycemic status with cigarette smoking outcomes among adults aged 20 years or older, United States, 2011–2023.

Predictor	Model 1, OR (95% CI)	Model 2, aOR (95% CI)	Model 3, aOR (95% CI)
Current smoking status			
No diabetes	1.00 (Reference)	1.00 (Reference)	1.00 (Reference)
Prediabetes	1.16 (1.02, 1.30)	1.23 (1.06, 1.42)	1.31 (1.10, 1.57)
Controlled diabetes	0.79 (0.68, 0.92)	0.83 (0.70, 0.99)	0.95 (0.76, 1.19)
Uncontrolled diabetes	1.17 (0.99, 1.39)	1.10 (0.88, 1.36)	1.37 (1.02, 1.83)

Quit attempts			
No diabetes	1.00 (Reference)	1.00 (Reference)	1.00 (Reference)
Prediabetes	1.21 (0.96, 1.52)	1.33 (1.00, 1.77)	1.43 (0.96, 2.14)
Controlled diabetes	0.90 (0.63, 1.28)	0.97 (0.65, 1.43)	1.05 (0.69, 1.61)
Uncontrolled diabetes	0.72 (0.50, 1.04)	0.73 (0.46, 1.15)	0.67 (0.38, 1.18)

Quit ratio			
No diabetes	1.00 (Reference)	1.00 (Reference)	1.00 (Reference)
Prediabetes	1.03 (0.90, 1.18)	0.82 (0.69, 0.97)	0.80 (0.66, 0.98)
Controlled diabetes	1.92 (1.65, 2.24)	1.37 (1.12, 1.66)	1.22 (0.96, 1.54)
Uncontrolled diabetes	1.15 (0.92, 1.43)	1.08 (0.80, 1.46)	0.81 (0.55, 1.21)

Note. Values are survey-weighted odds ratios with 95% confidence intervals. No diabetes was the reference category. Current smoking was assessed among all adults, past-year quit attempts among current smokers, and former smoking among ever smokers. Model 1 was unadjusted. Model 2 adjusted for age, sex, race and ethnicity, educational attainment, income-to-poverty ratio, and health-insurance status. Model 3 additionally adjusted for body mass index, alcohol consumption, depressive symptoms, and cardiometabolic comorbidity. Controlled diabetes was defined as diabetes with hemoglobin A1c below 7.0%, and uncontrolled diabetes as diabetes with hemoglobin A1c of 7.0% or higher. All models incorporated survey weights, strata, and primary sampling units.

Abbreviations: aOR, adjusted odds ratio; CI, confidence interval; HbA1c, hemoglobin A1c; OR, odds ratio.

For quit attempts, the overall glycemic-group association was significant in the fully adjusted model (*P* = 0.01), although none of the individual groups differed significantly from the no-diabetes reference group.

Among ever smokers, adults with prediabetes had 20% lower odds of being former rather than current smokers compared with adults without diabetes (aOR 0.80; 95% CI 0.66, 0.98). Controlled diabetes was associated with higher odds of former smoking in the unadjusted and partially adjusted models, but the association was no longer statistically significant after full adjustment (aOR 1.22; 95% CI 0.96, 1.54). Uncontrolled diabetes was not significantly associated with former-smoking status (aOR 0.81; 95% CI 0.55, 1.21) (Table 4).

## 4 Discussion

In this nationally representative analysis of US adults from NHANES 2011–2023, smoking burden and cessation indicators differed across the glycemic spectrum. After age standardization, current smoking prevalence was highest among adults with prediabetes and those with uncontrolled diabetes. In fully adjusted analyses, both groups had higher odds of current smoking than adults without diabetes. Prediabetes was also associated with lower odds of being a former rather than current smoker among ever smokers. Among current smokers, cigarette consumption and heavy smoking were descriptively higher in the diabetes groups. By contrast, differences in quit attempts were imprecise, and the higher crude quit ratio among adults with controlled diabetes was attenuated after adjustment. These results update earlier US surveillance studies showing that smoking remains common among adults with diabetes or prediabetes. Our study extends previous work by distinguishing prediabetes, controlled diabetes, and uncontrolled diabetes rather than treating diabetes as a single category. It also evaluates several complementary smoking measures, including current smoking, smoking intensity, quit attempts, and quit ratio. This approach provides a more detailed assessment of tobacco burden across the glycemic spectrum and identifies patterns that may be obscured in binary comparisons of adults with and without diabetes.

The higher age-adjusted smoking prevalence among adults with prediabetes and uncontrolled diabetes may reflect the co-occurrence of tobacco use with social and behavioral risk factors such as socioeconomic disadvantage, mental health conditions, and barriers to preventive care (Houle et al., 2016; Reid et al., 2010). Clinically, this matters because prediabetes represents a high-leverage prevention window. Smoking increases cardiovascular risk and may worsen insulin resistance, so cessation should be addressed alongside diet, physical activity, and weight management in patients with prediabetes. For patients with uncontrolled diabetes, continued smoking adds to already elevated baseline risk for cardiovascular and microvascular complications and may be a marker of unmet psychosocial needs that require more than brief advice.

Among current smokers, the higher number of cigarettes per day and the greater prevalence of heavy smoking in the diabetes groups suggest a higher degree of nicotine dependence, which could contribute to gaps in cessation outcomes. One potential mechanism is altered nicotine metabolism; prior work suggests nicotine metabolism may be higher in adults with type 2 diabetes, which could promote compensatory smoking and increase difficulty quitting (Keith et al., 2019). Diabetes-specific concerns may also interfere with sustained abstinence. Patients frequently cite fear of weight gain, worries about destabilizing glycemic control, diabetes distress, and competing self-management demands, all of which can make quitting harder even when motivation is present (Grech et al., 2024). These findings support a more intensive approach to cessation for smokers with diabetes, particularly those who smoke more heavily, including combined behavioral counseling and pharmacotherapy with proactive follow-up. In practice, tailoring can include anticipatory guidance about weight change, a plan for glucose monitoring and medication adjustment during a quit attempt, and linkage to diabetes education or behavioral health supports when distress or depression is prominent (Grech et al., 2023; Krist et al., 2021).

We also observed that uncontrolled diabetes had a higher smoking burden and the lowest quit attempts, whereas controlled diabetes had the highest quit ratio. Socioeconomic disadvantage, depressive symptoms, diabetes-related distress, competing self-management demands, and barriers to continuity of care may affect both glycemic management and the ability to quit smoking (Hill-Briggs et al., 2020; Wojujutari Ajele and Sunday Idemudia, 2025). In practice, this supports integrating tobacco treatment into routine diabetes care, with attention to mental health needs, affordability of pharmacotherapy, and planned follow-up.

A higher quit ratio in controlled diabetes may reflect greater cumulative cessation success over time. Patients with better glycemic control often have more consistent clinical contact, creating repeated opportunities for screening, counseling, and pharmacotherapy, which align with clinical guidance and are associated with higher treatment utilization in health systems (Grech et al., 2024; Geletko et al., 2022; Hwong et al., 2021). The quit ratio reflects cumulative cessation rather than recent motivation and increased among adults without diabetes, consistent with longer-term national declines in smoking prevalence (Creamer et al., 2019; Meza et al., 2023). The absence of clear improvement in dysglycemic groups may reflect persistent structural barriers and dependence-related challenges that limit sustained abstinence, underscoring the need to integrate evidence-based tobacco treatment into diabetes prevention and diabetes care as a core cardiometabolic risk-reduction strategy (VanFrank et al., 2024).

In trend analyses, age-standardized smoking prevalence declined among adults without diabetes and among those with prediabetes, whereas no clear linear declines were observed in controlled or uncontrolled diabetes. We also did not detect significant linear changes in quit attempts within glycemic groups over the cycles. These may reflect limited power for subgroup analyses and the likelihood that quit behavior changes nonlinearly over time.

Key strengths of this study included separating diabetes by glycemic control and evaluating multiple smoking metrics spanning exposure and cessation behavior. Several limitations should be considered. First, NHANES is cross-sectional, so we cannot assess sustained abstinence, relapse, or time since quitting; the quit ratio should be interpreted as a proxy for cumulative cessation rather than a longitudinal success rate. Second, smoking status and quit attempts are self-reported and may be misclassified. Third, glycemic classification may misclassify some participants, including those with undiagnosed diabetes. Fourth, models that adjust for downstream health variables may partially overadjust; therefore, estimates from parsimonious and fully adjusted models should be interpreted together. Although the study period spanned the COVID-19 pandemic, the analysis was not designed to estimate pandemic-attributable changes in smoking behavior because NHANES data collection was interrupted during the early pandemic. Future longitudinal studies should evaluate transitions in smoking behavior after diabetes diagnosis and changes in glycemic control, and should examine whether healthcare access, cessation pharmacotherapy use, and integrated chronic disease management explain differences across glycemic strata.

## 5 Conclusion

In US adults surveyed from 2011 to 2023, smoking prevalence remained high among individuals with prediabetes and uncontrolled diabetes, and smokers with diabetes reported higher smoking intensity. Differences in quit attempts and quit ratio suggest that barriers to cessation may be greatest in uncontrolled diabetes, whereas cumulative cessation may be more favorable in controlled diabetes. The results indicate that early interventions aimed at assisting individuals in quitting smoking prior to the onset of diabetes or maintaining control for patients with diabetes may lead to improved quit attempts. Therefore, these findings highlight the need to strengthen and tailor evidence-based cessation interventions within diabetes prevention and routine diabetes care, with particular attention to adults with prediabetes and uncontrolled diabetes to reduce cardiometabolic risk.

## CRediT authorship contribution statement

**Fuzhuo Wang:** Writing – review & editing, Writing – original draft, Visualization, Validation, Supervision, Software, Resources, Project administration, Methodology, Investigation, Funding acquisition, Formal analysis, Data curation, Conceptualization. **Jiashuang Xu:** Writing – review & editing, Visualization, Validation, Supervision, Software, Resources, Project administration, Methodology, Investigation, Funding acquisition, Formal analysis, Data curation, Conceptualization. **Yuxuan Ma:** Writing – review & editing, Visualization, Validation, Supervision, Software, Resources, Project administration, Methodology, Investigation, Funding acquisition, Formal analysis, Data curation, Conceptualization. **Jason Wong:** Writing – review & editing, Formal analysis, Data curation, Conceptualization. **Ligang Liu:** Writing – review & editing, Visualization, Validation, Supervision, Software, Resources, Project administration, Methodology, Investigation, Funding acquisition, Formal analysis, Data curation, Conceptualization. **Ye Huang:** Writing – review & editing, Visualization, Validation, Supervision, Software, Resources, Project administration, Methodology, Investigation, Funding acquisition, Formal analysis, Data curation, Conceptualization.

## Ethics declaration

### Informed consent and patient details

Written informed consent to take part in the study and to publish the article has been obtained from all participants or their legal representatives. The privacy rights of participants have been observed.

### Studies in human

This study was performed in compliance with relevant laws, regulatory frameworks and guidelines where the research took place.

The Research Ethics Review Board of the National Center for Health Statistics (NCHS) granted an exemption. The authors provided the following explanation: This study utilized de-identified, publicly available data from the National Health and Nutrition Examination Survey (NHANES). The NHANES protocol was approved by the National Center for Health Statistics (NCHS) Ethics Review Board (ERB), and all participants provided written informed consent. As this study is a secondary analysis of de-identified public-use data, it was determined to be exempt from additional institutional review board (IRB) review.

## Funding

No funding was obtained for this study.

## Declaration of competing interest

The authors declare that they have no known competing financial interests or personal relationships that could have appeared to influence the work reported in this paper.

## Data Availability

The study was conducted using NHANES data
