# Supplementary material for: Cigarette smoking and cessation indicators by glycemic status among adults in the United States, 2011–2023
Source: Prev Med Rep. 2026 Aug 19;70:103597. doi: 10.1016/j.pmedr.2026.103597 (PMC13521756; doi:10.1016/j.pmedr.2026.103597)
Supplement: Supplementary file 1 [file mmc1.docx]

**List of Supplementary Materials:**

**Table S1. Sample sizes, response rates, and analytic sample by survey cycle among adults aged 20 years or older in the National Health and Nutrition Examination Survey, United States, 2011-2023**

**Table S2. Operational definitions of glycemic status, cigarette smoking, and covariates used in the analysis of adults aged 20 years or older, National Health and Nutrition Examination Survey, United States, 2011-2023**

**Table S3. Bonferroni-adjusted pairwise post-hoc comparisons of cigarette smoking and cessation indicators across four glycemic status groups among adults aged 20 years or older, United States, 2011-2023**

**Table S4. Bonferroni-adjusted pairwise post-hoc comparisons of age-standardized cigarette smoking and cessation indicators across four glycemic status groups among adults aged 20 years or older, United States, 2011-2023**

Table S5. Age-standardized trends in current smoking, past-year quit attempts, and quit ratio by glycemic status among adults aged 20 years or older, United States, 2011-2023

**Table S1. Sample sizes, response rates, and analytic sample by survey cycle among adults aged 20 years or older in the National Health and Nutrition Examination Survey, United States, 2011-2023**

| Survey period | Eligible sample | Interviewed, n (%) | Examined, n (%) |
| --- | --- | --- | --- |
| 2011-2012 | 13431 | 9756 (72.64) | 9338 (69.53) |
| 2013-2014 | 14332 | 10175 (70.99) | 9813 (68.47) |
| 2015-2016 | 15327 | 9971 (65.06) | 9544 (62.27) |
| 2017-March 2020 | 27066 | 15560 (57.49) | 14300 (52.83) |
| August 2021-August 2023 | 22660 | 11933 (52.66) | 8860 (39.10) |

**Note.** Sampled, interviewed, and examined counts refer to the full National Health and Nutrition Examination Survey sample and are not restricted to adults aged 20 years or older. Interview and examination response rates are unweighted rates reported by the National Center for Health Statistics; they should not be recalculated by dividing the displayed interviewed or examined counts by the number of sampled persons because the official response-rate denominators account for survey eligibility and disposition.

**Table S2. Operational definitions of glycemic status, cigarette smoking, and covariates used in the analysis of adults aged 20 years or older, National Health and Nutrition Examination Survey, United States, 2011-2023**

| Measure | Operational definition | NHANES variable(s) |
| --- | --- | --- |
| Survey-defined diabetes | Self-reported clinician diagnosis (yes) or HbA1c ≥6.5% | Diabetes history item; laboratory HbA1c |
| No diabetes | No survey-defined diabetes and HbA1c <5.7% | Derived |
| Prediabetes | No survey-defined diabetes and HbA1c 5.7%–6.4% | Derived |
| Controlled diabetes | Survey-defined diabetes and measured HbA1c <7.0% | Derived |
| Uncontrolled diabetes | Survey-defined diabetes and measured HbA1c ≥7.0% | Derived |
| Never smoker | <100 cigarettes in lifetime | SMQ020 |
| Current smoker | ≥100 lifetime cigarettes and now smokes every day or some days | SMQ020 + SMQ040 |
| Former smoker | ≥100 lifetime cigarettes and now smokes not at all | SMQ020 + SMQ040 |
| Cigarettes/day | Average cigarettes/day on smoking days during past 30 days | SMD650 |
| Heavy smoking | Cigarettes/day ≥20 | Derived from SMD650 |
| Past-year quit attempt | Stopped smoking for >1 day in past 12 months because trying to quit | SMQ670 |
| Quit ratio | Former/(former + current) among ever smokers | Derived |
| Depressive symptoms | PHQ-9 score ≥10 | Derived |
| Alcohol use | Categorized into 4 groups based on average daily alcohol consumption with sex-specific cutoffs: Never drinkers (lifetime consumption <12 drinks); Mild (≤2 drinks/day in men, ≤1 drink/day in women); Moderate (3-4 drinks/day in men, 2-3 drinks/day in women); Heavy (≥5 drinks/day in men, ≥4 drinks/day in women) | ALQ101 + ALQ111 + ALQ130+RIAGENDR |
| Cardiometabolic comorbidity | Presence of any self-reported cardiometabolic condition: hypertension, high cholesterol, congestive heart failure, coronary heart disease, angina, myocardial infarction, or stroke. | BPQ020 + BPQ080 + MCQ160B + MCQ160C + MCQ160D + MCQ160E + MCQ160F |

**Note.** Controlled and uncontrolled diabetes describe glycemic status at the time of the examination because HbA1c was measured once. Derived measures were constructed consistently across included survey periods. HbA1c, hemoglobin A1c; NHANES, National Health and Nutrition Examination Survey; PHQ-9, nine-item Patient Health Questionnaire.

**Table S3. Bonferroni-adjusted pairwise post-hoc comparisons of cigarette smoking and cessation indicators across four glycemic status groups among adults aged 20 years or older, United States, 2011-2023**

| **Comparison** | **Current smoking, difference (%)** | **P** | **Quit attempt, difference (%)** | **P** | **Quit ratio, difference (%)** | **P** | **Cigarettes/day, mean difference (%)** | **P** | **Heavy smoking, difference (%)** | **P** |
| --- | --- | --- | --- | --- | --- | --- | --- | --- | --- | --- |
| Prediabetes vs no diabetes | 2.18 | 0.11 | 4.70 | 0.62 | 0.84 | 1.00 | 0.67 | 0.98 | 3.04 | 1.00 |
| Controlled diabetes vs no diabetes | -3.16 | <0.01 | -2.73 | 1.00 | 14.92 | <0.01 | 2.54 | 0.01 | 12.34 | <0.01 |
| Uncontrolled diabetes vs no diabetes | 2.38 | 0.47 | -8.11 | 0.43 | 3.33 | 1.00 | 2.21 | 0.05 | 10.85 | 0.16 |
| Controlled diabetes vs prediabetes | -5.33 | <0.01 | -7.43 | 0.63 | 14.08 | <0.01 | 1.87 | 0.18 | 9.30 | 0.04 |
| Uncontrolled diabetes vs prediabetes | 0.20 | 1.00 | -12.81 | 0.04 | 2.49 | 1.00 | 1.54 | 0.39 | 7.81 | 0.59 |
| Uncontrolled diabetes vs controlled diabetes | 5.53 | <0.01 | -5.38 | 1.00 | -11.59 | <0.01 | -0.33 | 1.00 | -1.49 | 1.00 |

**Note.** Differences were calculated as the estimate for the first glycemic group minus the estimate for the second group. Positive values indicate a higher estimate in the first group, and negative values indicate a lower estimate. Current smoking was assessed among all adults; past year quit attempts, cigarettes smoked per day, and heavy smoking were assessed among current smokers; and quit ratio was assessed among ever smokers. Heavy smoking was defined as 20 or more cigarettes per day. Quit ratio was calculated as the number of former smokers divided by the combined number of former and current smokers. Controlled diabetes was defined as diabetes with hemoglobin A1c below 7.0%, and uncontrolled diabetes as diabetes with hemoglobin A1c of 7.0% or higher. P values are two-sided and were adjusted for the six pairwise comparisons using the Bonferroni method.

**Table S4. Bonferroni-adjusted pairwise post-hoc comparisons of age-standardized cigarette smoking and cessation indicators across four glycemic status groups among adults aged 20 years or older, United States, 2011-2023**

| **Comparison** | **Current smoking, difference (%)** | **P** | **Quite attempt, difference (%)** | **P** | **Quit ratio, difference (%)** | **P** |
| --- | --- | --- | --- | --- | --- | --- |
| Prediabetes vs no diabetes | 4.63 | <0.01 | 8.16 | 0.14 | -7.44 | <0.01 |
| Controlled diabetes vs no diabetes | 2.50 | 1.00 | -0.93 | 1.00 | 2.46 | 1.00 |
| Uncontrolled diabetes vs no diabetes | 7.57 | 0.01 | -4.46 | 1.00 | -8.02 | 0.19 |
| Controlled diabetes vs prediabetes | -2.13 | 1.00 | -9.10 | 0.95 | 9.90 | 0.07 |
| Uncontrolled diabetes vs prediabetes | 2.94 | 1.00 | -12.62 | 0.12 | -0.58 | 1.00 |
| Uncontrolled diabetes vs controlled diabetes | 5.08 | 0.53 | -3.53 | 1.00 | -10.48 | 0.16 |

**Note.** Values are age-standardized percentage-point differences calculated as the estimate for the first glycemic group minus the estimate for the second group. Positive values indicate a higher estimate in the first group, and negative values indicate a lower estimate. Estimates were survey weighted and directly standardized to the 2000 United States standard population. Current smoking was assessed among all adults, past-year quit attempts among current smokers, and quit ratio among ever smokers. Quit ratio was calculated as the number of former smokers divided by the combined number of former and current smokers. Controlled diabetes was defined as diabetes with hemoglobin A1c below 7.0%, and uncontrolled diabetes as diabetes with hemoglobin A1c of 7.0% or higher. P values are two-sided and were adjusted for the six pairwise comparisons using the Bonferroni method.

Table S5. Age-standardized trends in current smoking, past-year quit attempts, and quit ratio by glycemic status among adults aged 20 years or older, United States, 2011-2023

|  | **2011-2012, % (SE)** | **2013-2014, % (SE)** | **2015-2016, % (SE)** | **2017-2020, % (SE)** | **2021-2023, % (SE)** | **P for Trend** |
| --- | --- | --- | --- | --- | --- | --- |
| **Current smoking** |  |  |  |  |  |  |
| No diabetes | 17.96 (1.34) | 19.87 (1.84) | 17.81 (0.98) | 16.37 (1.14) | 13.56 (1.43) | <0.01 |
| Prediabetes | 26.29 (2.72) | 22.37 (1.59) | 22.08 (2.6) | 20.69 (2.21) | 17.74 (1.51) | 0.01 |
| Controlled diabetes | 16.66 (3.87) | 23.76 (3.64) | 20.30 (4.73) | 16.62 (4.15) | 21.73 (3.76) | 0.63 |
| Uncontrolled diabetes | 27.88 (4.59) | 21.98 (3.78) | 24.27 (3.73) | 30.18 (6.31) | 13.76 (3.38) | 0.35 |
| **Past-year quit attempts** |  |  |  |  |  |  |
| No diabetes | — | 46.40 (2.85) | 49.89 (3.36) | 49.18 (2.6) | — | 0.72 |
| Prediabetes | — | 49.08 (5.53) | 57.06 (4.21) | 60.77 (5.31) | — | 0.09 |
| Controlled diabetes | — | 50.50 (7.52) | 55.57 (5.35) | 36.56 (6.17) | — | 0.98 |
| Uncontrolled diabetes | — | 44.02 (8.98) | 44.03 (6.27) | 44.75 (9.18) | — | 0.99 |
| **Quit ratio** |  |  |  |  |  |  |
| No diabetes | 55.90 (3.04) | 50.99 (2.86) | 55.87 (2.26) | 59.49 (2.07) | 60.82 (2.33) | 0.04 |
| Prediabetes | 43.8 7(3.14) | 49.12 (4.19) | 50.17 (3.84) | 49.66 (3.49) | 54.44 (3.5) | 0.16 |
| Controlled diabetes | 61.34 (8.53) | 41.49 (2.39) | 60.72 (5.81) | 65.11 (7.02) | 55.84 (5.5) | 0.63 |
| Uncontrolled diabetes | 37.72 (5.55) | 53.58 (7.16) | 52.78 (6.79) | 43.06 (6.33) | 65.00 (6.26) | 0.61 |

**Note.** Values are survey-weighted percentages with standard errors and were directly standardized to the 2000 United States standard population. Current smoking was assessed among all adults, past-year quit attempts among current smokers, and quit ratio among ever smokers. Quit ratio was calculated as the number of former smokers divided by the combined number of former and current smokers. Controlled diabetes was defined as diabetes with hemoglobin A1c below 7.0%, and uncontrolled diabetes as diabetes with hemoglobin A1c of 7.0% or higher. P values were obtained from design-adjusted Wald tests accounting for survey weights, strata, and primary sampling units. Dashes indicate that comparable quit-attempt data were unavailable for the survey period. SE, standard error.
